# Effects of mealworm (*Tenebrio molitor*) larvae hydrolysate on nutrient ileal digestibility in growing pigs compared to those of defatted mealworm larvae meal, fermented poultry by-product, and hydrolyzed fish soluble

**DOI:** 10.5713/ajas.19.0793

**Published:** 2019-12-24

**Authors:** Kyung Hoon Cho, Sun Woo Kang, Jong Sang Yoo, Dae Kil Song, Yi Hyung Chung, Gyoo Taik Kwon, Yoo Yong Kim

**Affiliations:** 1 Daehan feed Co., Ltd., R&D Center, Incheon 22300, Korea; 2 Department of Agricultural Biotechnology, College of Animal Life Sciences, Seoul National University, Seoul 08826, Korea; 3 Jeonbuk Institute for Food-Bioindustry, Jeonju 54810, Korea; 4 Berry & Biofood Research Institute, Gochang 56417, Korea

**Keywords:** T*enebrio Molitor*, Mealworm Larvae Hydrolysate, Ileal Amino Acid Digestibility, Growing Pigs

## Abstract

**Objective:**

To investigate effect of mealworm (*Tenebrio molitor*) larvae hydrolysate on nutrient ileal digestibility compared to those of dried mealworm larvae meal, fermented poultry by-product, and hydrolyzed fish soluble in growing pigs.

**Methods:**

A total of 12 crossbred ([Landrace×Yorkshire]×Duroc) growing pigs with average body weight of 28.70±0.32 kg were surgically equipped with simple T-cannulas. A total of 12 pigs were assigned to individual metabolic crates and allotted to one of four treatments with 3 replicates in a fully randomized design.

**Results:**

Apparent ileal digestibility (AID) of dry matter (DM) was the highest in pigs fed HML diet. AIDs of crude protein (CP) were higher in pigs fed HML and DMLM diets than those in pigs fed the other two diets. AID of total amino acid was higher (p = 0.06) in pigs fed HML diet. AIDs of lysine (Lys), methionine (Met), and threonine (Thr) were similar in pigs fed DMLM and HML diets, but were higher (p = 0.05, p<0.05, and p = 0.05, respectively) than those in pigs fed FPBM or HFS diet. Pigs fed HML diet had higher standardized ileal digestibilities (SIDs) of DM and CP (p<0.05 and p<0.05, respectively) compared to pigs fed the other FPBM and HFS diets. SIDs of total amino acid were not different (p = 0.06) between treatments. For SIDs of Lys, Met, and Thr, pigs fed HML and DMLM diets showed higher SIDs (p = 0.05, p<0.05, and p<0.05, respectively) than pigs fed FPBM and HFS diets. SIDs of non-essential amino acids (aspartic acid, glycine, and alanine) were higher (p<0.05, p< 0.05, and p<0.05, respectively) in pigs fed HML, FPBM, and DMLM diets than those in pigs fed the HFS diet. AID and SID of glutamic acid were higher in pigs fed HML and FPBM diets.

**Conclusion:**

In conclusion, dietary supplementation of mealworm larvae hydrolysate had higher digestibility in DM, CP, Lys, Met, and Thr compared to dietary supplementation with fermented poultry by-product and hydrolyzed fish soluble.

## INTRODUCTION

As the global population increase, there is a growing demand for meat production and the requirement of protein ingredients that are used to feed livestock also increase. However, traditional protein sources are no longer suitable to fully satisfy feed production growth in a sustainable way. Costs of soy meal and fish meal, environmental concerns, and diseases such as transmissible spongiform encephalopathy, foot and mouth disease, and avian influenza are drivers that lead to searches for alternative protein sources. The acceptance barrier may be lower for insects as food and feed. This is also reflected in the large number of trials on insects as sustainable protein-rich sources for aquaculture and livestock all over the world [1,2], including trials on the common housefly (*Musca domestica*), the black soldier fly (*Hermetia illucens*), the yellow mealworm (*Tenebrio molitor*), locusts (*Locusta migratoria*, *Schistocerca gregaria*, Oxya spec., *etc.*), and silkworms (*Bombyx mori*). Insects emit considerably less greenhouse gas than most livestock, requiring smaller spaces and having high fecundity with fast-growth. In addition, insects are rich in proteins, energy, fats, minerals, and vitamins [3,4]. In general, proteins contents in insects are 40% to 65%, similar to protein contents of fish meal, poultry meal, and meat meal [1,3]. However, insect proteins have higher utilization rates than proteins of these animal by-products [1]. Thus, insects are attracting attention as a substitute for fish meal, poultry meal, meat meal, and soybean meal [1,3,5].

Among many insects, mealworm ( *Tenebrio molitor*) larvae have attracted great attention. Dried mealworm larvae contain high amounts of crude protein (CP, 46% to 52%) and fat (25% to 35%) which contains abundant essential fatty acids with superior oxidative stability [1,6]. Studies on the feed value of mealworm larvae for swine production have been done [1,7,8]. Results have indicated that dried mealworm larvae powder is a potentially promising protein source.

Feed ingredient processing includes heat treatment, fermen tation, and enzymatic hydrolysis. These processes are used to increase the availability and reduce anti-nutritive factors of protein ingredients. In particular, hydrolysate contains a bioactive peptide having antimicrobial, antioxidant, and opioid-like effect. It has been focused as a strategy to optimize animal production because it contains short peptides and certain amino acids (AAs) (taurine, glycine [Gly], arginine, glutamic acid [Glu], and alanine [Ala]) that can enhance palatability. In addition, its small peptides can be readily absorbed in the small intestine. There have been several studies on nutritional values of fermented or hydrolyzed animal by-products to improve growth performance and nutrient digestibility [9–11]. Fermented fish meal was an effective protein ingredient to improve growth performance [9], and had higher apparent ileal digestibility of total AA than fish meal [11]. The hydrolyzed porcine intestinal mucosa product had similar to or greater degestible energy and metabolizable energy (ME) values than fish meal, and the standardized total track digestibility of phosphorus (P) was greater than fish meal [10]. However, there is no research on the efficacy of mealworm larvae hydrolysate as feedstock. We hypothesize that the enzymatic hydrolysis process could improve digestibility and have positive effects on peptide and AA absorption by decreasing protein molecular weight of mealworm larvae. Therefore, the objective of this study was to investigate nutrient ileal digestibility of mealworm larvae hydrolysate in growing pigs compared to those of defatted mealworm larvae meal, fermented poultry by-product, and hydrolyzed fish soluble in growing pigs.

## MATERIALS AND METHODS

### Animal care

The experimental procedure was approved by the Institutional Animal Care and Use Committee at Seoul National University (SNU-191007-9).

### Experimental animal and design

A total of 12 crossbred ([Landrace×Yorkshire]×Duroc) growing pigs with initial average body weight of 28.70±0.32 kg were surgically equipped with simple T-cannulas after they were deprived of feed for 24 h according to surgical procedures of Stein et al [12]. These pigs had a recovery period of two weeks. A total of 12 pigs were assigned to individual metabolic crates and allotted to one of four treatments (three replicates per treatment) in a fully randomized design. A total of 4 experimental diets were formulated using corn, soybean meal dextrin, palm kernel meal or wheat bran. Dietary treatments included the following: i) DMLM, the experimental diet which contained 10.0% defatted mealworm larvae meal (MPC, MILAE Bioresources Co., Ltd, Seoul, Korea); ii) HML, the experimental diet which contained 10% hydrolysate of mealworm (*Tenebrio molitor*) larvae; iii) FPBM, the experimental diet which contained 10% fermented poultry by-product (Neo-Pep, MOABIO Co., INC, Anyang, Korea); and iv) HFS, the experimental diet which contained 10% hydrolyzed fish soluble (FS Peptide, Sopropeche, Wimille, France). The defatted mealworm larvae meal, fermented poultry by-product, and hydrolyzed fish soluble are commonly available. They are used in the livestock feed industry. Chemical constituents of defatted mealworm larvae meal, hydrolysate of mealworm larvae, fermented poultry by-product and hydrolyzed fish soluble, and experimental diets are shown in Table 1 and 2.

### Preparation of hydrolysate

The 40 kg of defatted mealworm meal (MPC, MILAE Bioresources Co., Ltd, Korea) was mixed in 360 L of water at room temperature. The pH was adjusted to 6.5–7.0 using 1 N NaOH before adding enzymes. No later adjustment of pH was made. Enzymes used were Alcalase 2.4 AU-A/g and Flavourzyme 500 LAPU/g (Novo Nordisk A/S, Bagsvaerd, Denmark). Dosages of Alcalase and Flavourzyme were 20 mL and 20 g, respectively. Hydrolysis proceeded at 50°C for 3 hours with constant stirring at 40 rpm. Following hydrolysis, the hydrolyzed mixture was heated at 85°C for 30 min to inactivate enzymes followed by continuous centrifugation (MBPX810SGV-34CL, Alfa Laval, Lund, Sweden) at 9,000 rpm under 130 L/h flow rate condition. After centrifugation, microbial cells were removed with a vibrating membrane separation process (Pollsep VMF 400, Poll, Port Washington, NY, USA). Following vibrating filtration, ultrafiltration was performed to obtain a hydrolysate having a molecular weight of 100 kDa or less (ultrafiltration: MU-50000, Sartorius, Goettingen, Germany; filter model:Sartocon Cassette Hydrosart 100 kDa 0.6 m^2^, Sartorius, Germany). To facilitate handling, the hydrolysate was mixed with malto-dextrin in a ratio of 1:1 and then dried in a freezing dryer.

### Solubility and *in-vitro* digestibility

Nitrogen solubilities of hydrolysate of defatted mealworm larvae, defatted mealworm larvae meal, fermented poultry by-product, and hydrolyzed fish soluble were determined according to the method of Chobert et al [13]. Each 4 g of the sample mixed at 1:1 with dextrin was dispersed in 100 mL of distilled water followed by mixing with a Vortex for 10 min. The pH was adjusted to 3 with 2 N HCl. The mixtures were centrifuged at a maximum speed of 3,400 rpm (relative centrifugal force = 2,589 g) for 10 min at room temperature (HA-300, Hanil Industry Co, Deajeon, Korea). *In-vitro* CP digestibility was determined according to the method of Cho and Kim [14]. Nitrogen content of the supernatant was determined by using the Kjeldahl procedure with a Kjeltec (Kjeltec TM2200, Foss Tecator, Höganäs, Sweden) and CP content was then calculated (nitrogen×6.25; procedure 981.10; AOAC).

### Experimental diet and feeding

The experimental diets were formulated to contain defatted mealworm larvae meal, hydrolysate of mealworm, fermented poultry by-product, or hydrolyzed fish soluble at level of 10.0%. Chromic oxide was mixed to the diet at 0.5% as an indigestible marker to calculate digestibility. The calculated ME, CP, methionine (Met), lysine (Lys), calcium, and P requirement of experimental diets were adjusted according to requirements of NRC [15,16] and kept same level among the test diets. Raw materials, chemical composition, AAs, and essential fatty acids compositions of experimental diets are shown in Table 2. Raw materials and chemical composition of nitrogen-free diets are shown in Table 3. For N-free diets, protein level was adjusted to the minimum with corn starch as base. Each pig was fed 1.15 kg of experimental diet two times a day at 07:00 and 19:00 h. Experimental diet provided 2.86 times of the maintenance energy requirement (MEn = 106 kcal ME/kg^0.75^ [15,16]). Water was available throughout the experimental period via a drinking nipple.

### Sample collection

Samples of ileal digested content were collected between 8:00 and 20:00 h for two days after five days of adaptation. Ileal digesta were collected into plastic bags attached to cannulas and emptied into plastic containers containing ice every 20 minutes. All samples were immediately transferred after sampling and stored in a −20°C deep freezer to prevent changes in AAs sequence due to microbes until analysis. Samples were dried in a freezing dryer and finely ground to pass through a 1-mm screen for chemical analysis including moisture, CP, AAs, crude fat (Cfat), and fatty acids contents.

### Chemical analysis

Diets and collected samples were grounded with a Cyclotec CT 193 Sample Mill (Foss Tecator, Hillerod, Denmark) and then analyzed. Analysis of dry matter (DM) was conducted according to AOAC Method 967.03. Nitrogen content was analyzed using the Kjeldahl procedure with a Kjeltec (Kjeltec TM2200, Foss Tecator, Sweden) and CP content was then calculated (nitrogen×6.25; procedure 981.10; AOAC). Cfat contents and fatty acid contents were analyzed according to Soxhlet method (AOAC Method 932.02) and BF3 methanol gas chromatography method (Trace GC, Thermo, Göteborg, EU; AOAC Method 940.28), respectively. For the analysis of AAs except for Met and cysteine, diets and samples were hydrolyzed in 6 N HCl at 110°C for 24 hours (AOAC Method 999.13). The Met and cystine were determined after cold performic acid oxidation overnight and hydrolyzed with 7.5 N HCl (AOAC method 994.12). Individual AA was measured using an AA analyzer (Beckman 6300 Amino Acid Analyzer; Beckman Instruments Corp., Palo Alto, CA, USA). Chromium concentrations were determined using Inductively coupled plasma atomic emission spectroscopy (ICP-OES) (Thermo SCIENTIFIC, Waltham, MA, USA).

### Calculations

Standardized ileal digestibilities (SIDs) of CP, Cfat, AAs, and fatty acids were calculated according to the method described by Stein [17,18]. Basal endogenous losses of CP and AAs were measured using samples collected at the end of the ileum after feeding with non-nitrogen feed. Calculation of digestibility was done based on relative chromium concentrations of feed and ileal samples. Apparent ileal digestibility (AID) and SID were calculated using the following equations:

$$\begin{array}{l} \text{AID }(\%)=100-[(\text{ND}/\text{NF})\times (\text{CrF}/\text{CrD})\times 100]\\ \text{Basal endogenous AA losses }(\text{EAL},\text{mg}/\text{kg})=[\text{ND}\times (\text{CrF}/\text{CrD})]\\ \text{SID }(\%)=[\text{AID}+(\text{EAL}/\text{NF})]\times 100 \end{array}$$

Where ND (mg/kg), NF (mg/kg), CrF (mg/kg), and CrD (mg/kg) were nutrient concentration in the ileum digesta sample, nutrient concentration in the feed, chromium concentration in the feed, and chromium concentration in the ileum digesta sample, respectively.

### Statistical analysis

The data for AID and SID of nutrients were statistically analyzed in a randomized complete block design using the general linear model procedure of SAS (SAS Inst. Inc., Cary, NC, USA). The model included dietary treatment as independent variable. Each pig was considered as the experimental unit. The differences among means were declared significant at p<0.01 and p<0.05. When the significance was declared, the fisher’s least significance difference method was used to separate means. Variability in the data was expressed as standard error of means.

## RESULTS

Nutrient contents of defatted mealworm larvae meal, mealworm larvae hydrolysate, fermented poultry by-product, and hydrolyzed fish soluble are summarized in Table 1. These data indicated that defatted mealworm larvae meal had 36.76 g/16 g nitrogen essential AAs, which was higher than other protein sources. The hydrolysate of mealworm larvae had the highest content (at 10.36 g/16 g nitrogen) of Lys, an essential AA, followed by hydrolyzed fish soluble. The hydrolyzed mealworm larvae also had the highest content of Met+cystine (3.77 g/16 g nitrogen), followed by defatted mealworm larvae meal (2.94 g/16 g nitrogen). Protein and ash contents of hydrolysate of mealworm larvae were 58.88% and 14.90%, respectively. Results of nitrogen solubility and *in-vitro* digestibility analysis are shown in Table 4. The solubility and digestibility of mealworm larvae hydrolysate were the highest (85.71% and 98.96%, respectively), followed by those of fermented poultry by-product and hydrolyzed fish soluble. The nitrogen solubility and *in-vitro* digestibility of defatted mealworm larvae meal were the lowest (16.23% and 50.11%, respectively).

Results of AID are presented in Table 5. The AID of DM in pigs fed HML diet was the highest (89.47%), which was significantly (p<0.05) higher than those in pigs fed DMLM, FPBM, and HFS diets. AIDs of CP in pigs fed HML and DMLM diets were 89.31% and 86.37%, respectively. They were significantly (p<0.05) higher than those in pigs fed FPBM and HFS diets. AID of total AA in pigs fed HML diet was 79.52%, higher (p = 0.06) than AIDs in pigs fed FPBM and DMLM diets. AIDs of Lys, Met, and threonine (Thr) were similar in pigs fed DMLM and HML diets. They were higher (p = 0.05, p<0.05, and p = 0.05, respectively) than those in pigs fed FPBM and HFS diets. There were no significant differences in AIDs of other essential AAs. AIDs of non-essential AAs (aspartic acid [Asp], Gly, and Ala) were higher (p<0.05, p<0.05, and p<0.05, respectively) in pigs fed HML, FPBM, and DMLM diets than in pigs fed HFS diet. In particular, AIDs of Glu, a palatability enhancer, in pigs fed HML and FPBM diets were 78.98% and 77.07%, respectively. They were higher (p<0.05) than that in pigs fed the DMLM diet (72.71%).

Compared to pigs fed FPBM and HFS diets, pigs fed HML diet had higher (p<0.05 and p<0.05, respectively) SID of DM (93.31%) and CP (93.15%) (Table 6). In comparison between HML and DMLM diets, pigs fed HML diet showed higher digestibility, although the difference between the two was not statistically significant. In the case of SIDs of total AAs, although there was no difference between treatments (p = 0.06), pigs fed HML showed higher digestibility, followed by those fed with FPBM and DMLM diets. Regarding SIDs of Lys, Met, and Thr, pigs fed HML and DMLM diets showed higher SIDs (p = 0.05, p<0.05, and p<0.05, respectively) than pigs fed FPBM and HFS diets. There were no significant differences in SIDs of other essential AAs. Same as the trend of AID, SIDs of non-essential AAs (Asp, Gly, and Ala) were higher (p<0.05, p<0.05, and p<0.05, respectively) in pigs fed HML, FPBM, and DMLM diets than in pigs fed HFS diet. SIDs of Glu, a palatability enhancer, in pigs fed HML and FPBM diets were 82.82% and 80.91%, higher p<0.05) than that in pigs fed the DMLM diet. There were no significant differences in AID or SID digestibility of fatty acids except for those in pigs fed the DMLM diet.

## DISCUSSION

Traditional protein sources used as feedstock have been proteins of animal or plant origin. However, interest in insect proteins as an alternative protein source that can be grown on organic streams has increased and the test have been conducted to compare insect proteins with several feed ingredients, such as fish meal, casein, spray-dried porcine plasma, soybean meal, and wheat gluten meal [19,20]. In the present experiment, we used hydrolysate of mealworm larvae, defatted mealworm larvae meal, and two processed animal by-products protein to compare their nutrient digestibilities in growing pigs.

In general, dried mealworm larvae contain high amounts of CP (46% to 52%) and fat (25% to 35%) with relatively low ash content (3% to 5%) [3,21,22]. When dried mealworms are defatted through the degreasing process, CP content ranges from 65% to 70% [3,23–25]. In this experiment, CP and essential AA content in defatted mealworm larvae meal were 68% and 36.8 (g/16 g nitrogen) respectively, and the essential AA content was higher than those of other three proteins. The CP content was calculated by multiplying the nitrogen content by a factor of 6.5, which was not an accurate method for insect proteins because mealworm larvae proteins were mainly composed of proteins derived from the exoskeleton, chiefly cuticular non-protein nitrogen known to contain chitin, a linear polymer of β-(1–4) N-acetyl-D-glucosamine units with a chemical structure similar to that of cellulose [20,26]. When the CP content of mealworm larvae having exoskeleton was calculated using a factor of 6.25, it could be overestimated by about 20%. Therefore, a factor of 4.75 has been proposed for calculating the CP content of whole mealworm larvae having exoskeleton and a factor of 5.6 has been proposed for calculating aqueous extracted protein [27]. However, the defatted mealworm larvae meal used in this experiment was not only high in CP, but also high in essential AAs contents, which were comparable to those of other processed animal proteins. Contents of essential AAs Lys and Met+cystine in the defatted meal worm larvae meal were 5.82 g and 2.94 g per 16 g of nitrogen, respectively, similar to results of a previous study [26]. The CP content in mealworm larvae hydrolysate was 58.9%. Although moisture content was corrected to 5%, it was about 62.0%, lower than those of other processed proteins used in this experiment and 74% of protein content obtained after the mealworm larvae was precipitated with acid (pH = 4) and extracted with water [28]. In this experiment, ultrafiltration was performed to obtain a hydrolysate having a molecular weight of 100 kDa or less. Relatively large molecular weight non-protein nitrogen might not be included in the hydrolysate. This was considered to be the reason why the CP content in the hydrolysate obtained in this experiment was low. Although CP and total essential AA contents in hydrolysate of mealworm were not higher than those in other processed animal proteins, Lys and Met+cystine contents were the highest (10.4 g and 3.8 g per 16 g nitrogen, respectively) in the hydrolysate of mealworm. Values of essential AA (Lys, Met+cysteine, and Thr) index (EAAI) [29] for defatted mealworm larvae meal, hydrolyzed mealworm larvae, fermented poultry by-product, and hydrolyzed fish soluble were 0.51, 0.49, 0.40, and 0.44, respectively (AA requirements based on Daehan Feed Co. data). EAAI values of defatted mealworm larvae and hydrolyzed mealworm larvae were similar to each other, but higher than those of fermented poultry by-product and hydrolyzed fish soluble. The ash content of mealworm larvae hydrolysate was higher than those (from about 4.0% to 12.0%) of hydrolysate of poultry viscera (8.8%), fish protein concentrates (6.0%) and isolates (4.0%), fermented fish silage (4.6%) and hydrolyzed porcine intestinal mucosa product (10.0% and 12.0%) [10,30–32]. There was no process to increase ash content in enzymatic hydrolysis. However, the ash content was higher than that of the feed ingredient (defatted mealworm meal, 8%) used for hydrolysis in the present study. The ash content of the raw material has a great effect on energy evaluation. Thus, it should be reconfirmed in future studies in terms of feed ingredient value.

Digestibility was measured when experimental diets con taining 25% to 35% plant-derived protein ingredients were fed to pigs ranging from 18 kg to 59 kg body weight [33–35]. The CP AID of diets including Chinese, Argentine, and Korean soybean meal were 70.34%, 71.30%, and 76.57% respectively, while that of diet including rapeseed meal was 66.34% in pigs with body weight of 58.6 kg [33]. The CP, Lys, and Met SID of diet including soybean meal were 84.03%, 85.02%, and 87.27%, respectively, in pigs with body weight of 18.2 kg [34]. AID of CP, Lys, and Met of diet including canola meal were 62.0%, 72.0%, and 77.4%, respectively, in pigs with body weight of 35.0 kg [35]. The CP AID and SID of DMLM, HML, FPBM, and HFS diets were similar to or higher than those of diets including plant protein ingredients, such as soybean meal, rapeseed meal and canola meal. Based on reviews and the result that AID and SID of experimental diets were equal to or higher than those of diets including several plant protein ingredients, it can be inferred that digestibilities of the defatted mealworm larvae meal, hydrolysate of mealworm, hydrolyzed poultry by-product, and fermented fish soluble used in this experiment could be generally higher than those of plant protein ingredients. Jin et al [36] have shown that the total track digestibilities of CP were 92.17% and 93.04% in pigs fed diets including 4.5% and 6.0% dried mealworm larvae, respectively. Although they [36] measured total digestibility in younger pigs than those of our study, the result showed higher digestibility than AID (86.37%) and SID (90.21%) in the present study due to differences in experimental diet composition and lower feeding amount (2% of body weight) in their study than that of this study (4% of body weight). Defatted mealworm larvae meal had the lowest solubility in 2 N HCl solution and *in-vitro* digestibility, however CP AID and SID in pigs fed DMLM diet were similar to those in pigs fed FPBM and HFS diets. It could be said that the digestibility of the DMLM diet was not negatively affected by the defatted mealworm larvae meal at 10% usage level.

Insect proteins may also have increased availability when processed. Although there are studies on the physiological activity of low molecular weight mealworm larvae peptide in the field of food [22,26], few studies have evaluated mealworm larvae peptide as a protein source in pig nutrition when processed. In comparison between HML and DMLM diets, DM AID of HML diet was 89.47%, higher than that of DMLM diet (87.45%). AIDs of CP and total AA and SIDs of DM, CP, and total AA tended to be higher in HML diet than those in DMLM diet, although there was no statistically significant difference between the two diets. The lower nitrogen solubility in 2 N HCl solution and CP digestibility of DMLM diet might have attributed to indigestibility of chitin-N and/or acid detergent fiber bound-N. Chitin, a linear polymer of β-(1–4) N-acetyl-D-glucosamine units with a chemical structure similar to that of cellulose, is combined with protein and distributed widely on shell of crabs, shrimp and insects. It is soluble in a narrow range of HCl, carboxylic and sulfonic acids, namely, formic, di- and trichloroacetic and methanesulfonic acids and slowly degraded. It is distributed in the form of chitin-protein complexes as a major component of the cuticle layer of mealworm larvae [20,26]. When proteins are hydrolyzed to low molecular weight peptides, they can be easily absorbed into the body with high utilization rates. Solubility and *in-vitro* digestibility of hydrolysate of defatted mealworm larvae meal were 85.71% and 98.96%, respectively, higher than those of defatted mealworm (*Tenebrio molitor*) larvae meal, fermented poultry by-product and hydrolyzed fish soluble. The higher solubility and digestibility suggested extensive hydrolysis of mealworm larvae into short peptides, which were more soluble compare to intact and the other two processed animal protein sources. We used a combination of Alcalase (endo-peptidase) and Flavourzyme (exo-peptidase) which have similar optimum activity temperature and pH conditions [37]. When mealworm larvae protein was hydrolyzed using Alcalase and Flavourzyme, it was hydrolyzed to less than 100 kDa [22]. Hydrolyzed low molecular weight peptides could be rmore eadily absorbed than raw mealworm protein. Although only proteins having a molecular weight of 100 kDa or less were measured, the recovery rate was 13.8% (data not shown), higher than that (9.97%) in experiment using enzymatic hydrolyzed poultry meal [37]. However, this recovery rate was lower than that in other study [22] using hydrolyzed mealworm larvae protein. It takes about 6 to 8 hours for mealworms to be fully hydrolyzed using Alcalase and Flavorzyme [22]. However, in the present study, hydrolysis was performed for only 3 hours in consideration of industrial economics, so the recovery rate was low.

In pigs fed the HML diet, since the hydrolysate of meal worm larvae was composed only of peptides with molecular weight of 100 kDa or less, AID and SID of DM and CP were higher than those of FPBM and HFS diets. In another study [38], the CP AID of hydrolysed fish soluble was 87.0%, higher than results of the present study. AID and SID of test diets including mealworm larvae-derived ingredients were higher than those of FPBM and HFS diets. This meant that the digestibility of mealworm protein was high regardless of processing. Thus, it had a relatively high value as a protein ingredient. The quality of hydrolysed fish soluble may vary depending on the composition, type, and fish species of raw materials used for hydrolysis. However, hydrolysate of mealworm larvae may have less variation in quality because it used relatively less varied mealworm larvae meal.

Results of this study showed that hydrolyzed mealworm (*Tenebrio molitor*) larvae would be a suitable ingredient in growing pigs. It is especially valuable from the stand-point of CP and essential AA ileal digestibility. In younger pigs, hydrolysate of mealworm larvae could replace fermented poultry by-product and hydrolyzed fish soluble. High CP and essential AA ileal digestibility of mealworm larvae hydrolysate containing diet may act on the deposition of AAs in pig meat and the growth performance of growing pigs. Further studies are needed to explore this possibility. In addition, research in the area of mealworm larvae hydrolysate may lead to discovery of novel peptide sequences that might be more potent and/or more bioavailable than bioactive peptides generated from more conventional dietary proteins.

## CONCLUSION

In conclusion, dietary supplementation of mealworm larvae hydrolysate had higher digestibility in DM, CP, Lys, Met, and Thr compared to dietary supplementation with fermented poultry by-product and hydrolyzed fish soluble. The nitrogen soulubility and *in-vitro* CP digestibility of mealworm larvae hydrolysate were also high. Thus, mealworm larvae hydrolysate can be a potentially attractive alternative protein-rich feed ingredient in growing pigs.

## Figures and Tables

**Table 1:** Composition of defatted mealworm larvae meal, hydrolysate of mealworm larvae, fermented poultry by-product and hydrolyzed fish soluble, as-fed basis

Chemical composition1)	Defatted mealworm larvae meal2)	Hydrolysate of mealworm larvae3)	Fermented poultry by-product4)	Hydrolyzed fish soluble5)
Moisture (%)	8.50	10.58	5.83	5.38
Crude protein (%)	68.00	58.88	71.52	89.04
Crude fat (%)	4.00	0.26	11.22	7.00
Crude ash (%)	8.00	14.90	9.30	5.00
Ca (%)	0.60	0.20	0.10	0.20
Total phosporus (%)	0.50	0.82	1.00	0.75
Amino acid6)				
Essential	36.76	30.05	31.44	19.24
Methionine	2.07	3.02	1.96	1.36
Cystine	0.87	0.75	0.67	0.15
Valine	4.78	3.02	3.89	1.80
Isoleucine	3.41	1.80	2.96	1.47
Leucine	7.07	3.77	6.09	2.90
Phenylalanine	3.74	2.07	3.44	1.76
Tyrosine	3.41	1.15	2.92	0.00
Histidine	1.49	0.92	2.89	1.52
Lysine	5.82	10.36	3.12	6.00
Threonine	4.10	3.19	3.50	2.28
Non-essential	45.99	33.17	47.81	49.04
Serine	6.18	3.19	3.80	3.89
Arginine	6.01	3.19	5.86	4.93
Glutamic acid	13.46	10.39	13.25	7.95
Aspartic acid	7.66	4.82	7.54	5.33
Proline	1.66	3.26	4.98	6.79
Glycine	6.12	4.48	6.61	13.98
Alanine	4.90	3.84	5.77	6.17

1) Lab. of Daehanfeed Co. LTD.

2) Defatted mealworm (*Tenebrio molitor*) larvae meal: MPC, MILAE Bioresources Co., Ltd, Seoul, Korea.

3) Hydrolysate of mealworm (*Tenebrio molitor*) larvae.

4) Fermented poultry by-product: Neo-Pep, MOABIO Co., INC, Anyang, Korea.

5) Hydrolyzed fish soluble: FS Peptide, Sopropeche, Wimille, France.

6) g/16 g nitrogen.

**Table 2:** Composition of the experimental diets, as-fed basis

Items	Treatments1)			

	DMLM	HML	FPBM	HFS
Ingredients (%)				
Ground corn	65.01	54.81	62.54	67.44
Soybean meal, 45%	6.36	8.43	3.63	0.43
Defatted mealworm larvae meal	10	0	0	0
Hydrolysate of mealworm larvae	0	10	0	0
Fermented poultry by-product	0	0	10	0
Hydrolyzed fish soluble	0	0	0	10
Palm kernel meal	4	10	4	0
Wheat bran	0	0	5	7
Dextrin	10	10	10	10
Tallow	0.75	3.37	0.46	0.97
MDCP	1.58	1.28	1.27	1.49
Limestone	0.8	0.92	1.02	0.94
DL-methionine, 99%	0.05	0	0.14	0.1
L-threoninem 99%	0.09	0.19	0.29	0.26
L-lysine-HCl, 78%	0.36	0	0.65	0.37
Salt	0.3	0.3	0.3	0.3
Vitamin premix2)	0.1	0.1	0.1	0.1
Mineral premix3)	0.1	0.1	0.1	0.1
Cr_2_O_3_	0.5	0.5	0.5	0.5
Sum	100	100	100	100
Chemical composition4)				
ME (kcal/kg)	3,265	3,265	3,265	3,265
Crude protein (%)	15.69	15.69	15.69	15.69
Crude fat (%)	3.69	5.83	4.09	4.19
Crude fiber (%)	2.17	2.78	2.44	2.08
Lysine (%)	1.02	1.02	1.02	1.02
Methionine (%)	0.35	0.35	0.35	0.35
Threonine (%)	0.68	0.68	0.68	0.68
Ca (%)	0.66	0.66	0.66	0.66
Total P (%)	0.56	0.56	0.56	0.56
Essential fatty acid5)				
Palmitic acid	16.85	19.71	18.54	19.84
Stearic acid	4.47	8.10	5.01	6.70
Oleic acid	27.84	32.99	31.19	32.78
Linoleic acid	39.34	24.49	35.55	36.66
Linolenic acid	1.60	1.05	1.39	1.53

MDCP, mono-di-calcium phosphate (Ca, 17.6% and total P, 20.4%).

1) DMLM, the experimental diet which contained 10.0% defatted mealworm (*Tenebrio molitor*) larvae meal (MPC, MILAE Bioresources Co., Ltd, Seoul, Korea); HML, the experimental diet which contained 10% hydrolysate of mealworm (*Tenebrio molitor*) larvae; FPBM, the experimental diet which contained 10% fermented poultry by-product (Neo-Pep, MOABIO Co., INC, Anyang, Korea); and HFS, the experimental diet which contained 10% hydrolyzed fish soluble (FS Peptide, Sopropeche, Wimille, France).

2) Provided the following per kilogram of diet: vitamin A, 12,000 IU as vitamin A acetate; vitamin D_3_, 2,000 IU; vitamin E, 40 IU as dl-α-tocopheryl acetate; biotin, 0.3 mg as d-biotin; riboflavin, 5.0 mg; thiamine 3 mg as thiamine mononitrate; pyridoxine 5 mg as pyridoxine hydrochloride; folic acid 0.5 mg; pantothenic acid, 15 mg as d-calcium pantothenate; niacin, 30 mg as nicotinic acid; vitamin B_12_, 50 μg as cyanocobalamin; and vitamin K_3_, 2.0 mg as menadione nicotinamide bisulfate.

3) Provided the following per kilogram of diet: Cu 15 mg as CuSO_4_H_2_O; Fe 120 mg as FeSO_4_H_2_O; Zn 95 mg as ZnSO_4_H_2_O; Mn 40 mg as MnSO_4_H_2_O; I 1.0 mg as Ca(IO_3_)H_2_O; Co 0.5 mg as CoSO_4_H_2_O; and Se 0.3 mg as NaSeO_3_H_2_O.

4) Calculated values.

5) Measured values.

**Table 3:** Composition of the nitrogen-free diets, as-fed basis

Items	
Ingredient (%)	
Corn starch	84.02
Cellulose	6.92
MDCP	2.67
Limestone	0.39
Salt	0.30
Vitamin premix1)	0.10
Mineral premix2)	0.10
Cr_2_O_3_	0.50
Sum	100.00
Chemical composition3)	
ME (kcal/kg)	3,265
Crude protein (%)	15.69
Lysine (%)	0.00
Methionine (%)	0.00
Threonine (%)	0.00
Ca (%)	0.66
Total P (%)	0.56

MDCP, mono-di-calcium phosphate (Ca, 17.6% and total P, 20.4%); ME, metabolizable energy.

1) Provided the following per kilogram of diet: vitamin A, 12,000 IU as vitamin A acetate; vitamin D_3_, 2,000 IU; vitamin E, 40 IU as dl-α-tocopheryl acetate; biotin, 0.3 mg as d-biotin; riboflavin, 5 mg; thiamine 3 mg as thiamine mononitrate; pyridoxine 5 mg as pyridoxine hydrochloride; folic acid 0.5 mg; pantothenic acid, 15 mg as d-calcium pantothenate; niacin, 30 mg as nicotinic acid; vitamin B_12_, 50 μg as cyanocobalamin; and vitamin K_3_, 2.0 mg as menadion nicotinamide bisulfate.

2) Provided the following per kilogram of diet: Cu 15 mg as CuSO_4_H_2_O; Fe 120 mg as FeSO_4_H_2_O; Zn 95 mg as ZnSO_4_H_2_O; Mn 40 mg as MnSO_4_H_2_O; I 1.0 mg as Ca(IO_3_)H_2_O; Co 0.5 mg as CoSO_4_H_2_O; and Se 0.3 mg as NaSeO_3_H_2_O.

3) Calculated values.

**Table 4:** Nitrogen solubility and in-vitro crude protein digestibility of defatted mealworm larvae meal, hydrolysate of mealworm larvae, fermented poultry by-product and hydrolyzed fish soluble

Items	Defatted mealworm larvae meal1)	Hydrolysate of mealworm larvae2)	Fermented poultry by-product3)	Hydrolyzed fish soluble4)	SEM	p-value
2 N HCl solubility5)	16.23^d^	85.71^a^	68.82^b^	41.88^c^	0.39	<0.01
*In-vitro* digestibility6)	50.11^d^	98.96^a^	93.77^b^	82.78^c^	0.28	<0.01

SEM, standard error of the mean.

1) Defatted mealworm (*Tenebrio molitor*) larvae meal: MPC, MILAE Bioresources Co., Ltd, Seoul, Korea.

2) Hydrolysate of mealworm (*Tenebrio molitor*) larvae.

3) Fermented poultry by-product: Neo-Pep, MOABIO Co., INC, Anyang, Korea.

4) Hydrolyzed fish soluble: FS Peptide, Sopropeche, Wimille, France.

5) Method described by Chorbert et al [18].

6) Method described by Cho and Kim [19].

a–d Means in a same row with different superscript are significantly different (p<0.01).

**Table 5:** Effect of protein ingredient on apparent ileal digestibility in growing pigs

Items	Treatments1)				SEM	p-value

	DMLM	HML	FPBM	HFS		
Dry matter (%)	87.45^b^	89.47a	87.05^b^	85.88^b^	0.462	<0.01
Crude protein (%)	86.37^ab^	89.31^a^	85.55^b^	83.41^b^	0.942	0.02
Crude fat (%)	82.12^b^	89.80^a^	89.03^a^	88.31^a^	1.119	<0.01
Total amino acid (%)	78.09	79.52	78.17	75.35	0.845	0.06
Essential amino acid (%)						
Lysine	79.68^ab^	79.92^a^	78.87^bc^	78.74^c^	0.269	0.05
Methionine	79.78^a^	79.93^a^	79.17^b^	79.08^b^	0.160	0.02
Threonine	79.36^ab^	79.92^a^	78.94^b^	78.83^b^	0.224	0.05
Valine	76.76	78.26	78.87	69.69	2.261	0.09
Isoleucine	76.45	78.02	78.98	72.62	1.658	0.13
Leucine	77.17	78.27	78.88	75.81	1.017	0.25
Phenylalanine	76.69	78.28	78.78	77.01	0.888	0.37
Histidine	76.91	78.78	78.83	74.74	1.528	0.28
Arginine	75.70	79.56	78.36	78.01	1.655	0.47
Non-essential amino acid (%)						
Aspartic acid	74.83^a^	78.36^a^	77.74^a^	68.06^b^	1.895	0.03
Serine	74.99	78.55	79.01	67.85	2.774	0.09
Glutamic acid	72.71^bc^	78.98^a^	77.07^ab^	69.88^c^	1.319	0.01
Glycine	65.32^a^	76.83^a^	76.35^a^	39.53^b^	4.709	<0.01
Alanine	76.17^a^	78.60^a^	77.74^a^	65.96^b^	1.385	<0.01
Tyrosine	74.70	77.44	78.54	77.58	1.350	0.31
Proline	74.90	79.20	76.79	63.88	4.532	0.18
Fatty acid (%)						
Palmitic acid	72.67^b^	79.74^a^	79.05^a^	79.67^a^	0.928	<0.01
Stearic acid	72.35^b^	79.63^a^	78.87^a^	79.66^a^	0.972	<0.01
Oleic acid	73.80^b^	79.89^a^	79.39^a^	79.93^a^	0.915	<0.01
Linoleic acid	72.78^b^	79.83^a^	79.13^a^	79.51^a^	1.120	0.01
Linolenic acid	73.31^b^	79.81^a^	78.76^a^	79.12^a^	0.884	<0.01

SEM, standard error of means.

1) DMLM, the experimental diet which contained 10.0% defatted mealworm (*Tenebrio molitor*) larvae meal (MPC, MILAE Bioresources Co., Ltd, Seoul, Korea); HML, the experimental diet which contained 10% hydrolysate of mealworm (*Tenebrio molitor*) larvae; FPBM, the experimental diet which contained 10% fermented poultry by-product (Neo-Pep, MOABIO Co., INC, Anyang, Korea); and HFS, the experimental diet which contained 10% hydrolyzed fish soluble (FS Peptide, Sopropeche, Wimille, France).

a–c Means in a same row with different superscript are significantly different (p<0.05).

**Table 6:** Effect of protein ingredient on standardized ileal digestibility in growing pigs

Items	Treatments1)				SEM	p-value

	DMLM	HML	FPBM	HFS		
Dry matter (%)	91.29^ab^	93.31^a^	90.89^bc^	88.96^c^	0.462	0.02
Crude protein (%)	90.21^ab^	93.15^a^	89.39^b^	86.49^b^	1.081	0.04
Crude fat (%)	85.96^b^	93.64^a^	92.87^a^	91.39^a^	1.286	0.02
Total amino acid (%)	81.93	83.36	82.01	79.19	0.845	0.06
Essential amino acid (%)						
Lysine	83.52^ab^	83.76^a^	82.71^bc^	82.58^c^	0.269	0.05
Methionine	83.62^a^	83.77^a^	83.01^b^	82.92^b^	0.160	0.02
Threonine	83.20^ab^	83.76^a^	82.78^bc^	82.67^c^	0.224	0.02
Valine	80.60	82.10	82.71	73.53	2.261	0.09
Isoleucine	80.29	81.86	82.82	76.46	1.658	0.13
Leucine	81.01	82.11	82.72	79.65	1.017	0.25
Phenylalanine	80.53	82.12	82.62	80.85	0.888	0.37
Histidine	80.75	82.62	82.67	78.58	1.528	0.28
Arginine	79.54	83.40	82.20	81.85	1.655	0.47
Non-essential amino acid (%)						
Aspartic acid	78.67^a^	82.20^a^	81.58^a^	71.90^b^	1.895	0.03
Serine	78.83	82.39	82.85	71.69	2.774	0.09
Glutamic acid	76.55^bc^	82.82^a^	80.91^ab^	73.72^c^	1.319	0.01
Glycine	69.16^a^	80.67^a^	80.19^a^	43.37^b^	4.709	<0.01
Alanine	80.01^a^	82.44^a^	81.58^a^	69.80^b^	1.385	<0.01
Tyrosine	78.54	81.28	82.38	81.42	1.350	0.31
Proline	78.74	83.04	80.63	67.72	4.532	0.18
Fatty acid (%)						
Palmitic acid	76.71^b^	83.58^a^	82.89^a^	81.72^a^	1.097	0.01
Stearic acid	76.19^b^	83.47^a^	82.71^a^	81.72^a^	1.051	<0.01
Oleic acid	77.64^b^	83.73^a^	83.23^a^	81.99^a^	1.114	0.03
Linoleic acid	76.62^b^	83.67^a^	82.97^a^	81.56^a^	1.311	0.03
Linolenic acid	77.15^c^	84.41^a^	82.60^ab^	79.89^bc^	0.875	<0.01

SEM, standard error of means.

1) DMLM, the experimental diet which contained 10.0% defatted mealworm (*Tenebrio molitor*) larvae meal (MPC, MILAE Bioresources Co., Ltd, Seoul, Korea); HML, the experimental diet which contained 10% hydrolysate of mealworm (*Tenebrio molitor*) larvae; FPBM, the experimental diet which contained 10% fermented poultry by-product (Neo-Pep, MOABIO Co., INC, Anyang, Korea); and HFS, the experimental diet which contained 10% hydrolyzed fish soluble (FS Peptide, Sopropeche, Wimille, France).

a–c Means in a same row with different superscript are significantly different (p<0.05).
